# A multicenter, double‐blind, randomized, controlled study of patients with treatment‐resistant schizophrenia treated with yokukansan for 12 weeks

**DOI:** 10.1002/pcn5.155

**Published:** 2023-11-14

**Authors:** Jun Horiguchi, Rei Wake, Kenta Murotani, Haruo Seno, Tsuyoshi Miyaoka, Ken Inoue

**Affiliations:** ^1^ Department of Psychiatry Shimane University School of Medicine Izumo Japan; ^2^ Biostatistics Center Kurume University Kurume Japan; ^3^ Psychiatry Matsue Aoba Hospital Matsue Japan; ^4^ Research and Education Faculty, Medical Sciences Cluster, Health Service Center Kochi University Kochi Japan

**Keywords:** randomized controlled study, safety, schizophrenia, treatment, yokukansan

## Abstract

**Aim:**

We conducted a 12‐week double‐blind, placebo‐controlled, multicenter study to evaluate the efficacy and safety of yokukansan in patients with schizophrenia.

**Methods:**

Patients with schizophrenia resistant to antipsychotics whose Positive and Negative Syndrome Scale (PANSS) scores were stable within five points were enrolled and assigned to the yokukansan or placebo group. Fifty‐three of the 61 consenting patients were allocated to the yokukansan (*n* = 27) and placebo (*n* = 26) groups.

**Results:**

The changes in total and positive PANSS scores at 12 weeks were significantly greater in the yokukansan group than in the placebo group. There were no significant changes in other psychiatric symptom rating scores in either group. Adverse reactions were reported in six of 27 patients (22.2%) in the yokukansan group and five of 26 patients (19.2%) in the placebo group, all of which were nonserious.

**Conclusion:**

Yokukansan is very safe and has clinical potential as a treatment for schizophrenia in combination with Western medicine.

## Graphical abstract

In a 12‐week double‐blind, placebo‐controlled, multicenter study, yokukansan changes in total and Positive and Negative Syndrome Scale each score decreased. Yokukansan is very safe and has clinical potential as a treatment for schizophrenia in combination with Western medicine.

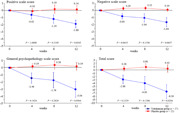

## INTRODUCTION

The number of patients in Japan with schizophrenia, including related diseases, is estimated to be approximately 792,000.^1^ The typical onset period is late adolescence or early adulthood. It is a severe mental disorder with various psychiatric symptoms that majorly impact society and individual patients.^2^ The core symptoms are categorized as positive (delusions, hallucinations, and loss of contact with reality), negative (impaired motivation, reduction in spontaneous speech, and social withdrawal), and cognitive aspects (impaired attention and memory, decline of working memory holding a limited amount of information for a short period and function to act and think based on it).^3^

For the treatment of schizophrenia, a combination of medication and psychosocial treatment is recommended, which can alleviate symptoms by modulating the action of neurotransmitters, such as dopamine, glutamate, and serotonin, in the central nervous system.^4^, ^5^ Discontinuing antipsychotics in patients who are stable during treatment increases the risk of relapse, and antipsychotics that can be administered continuously are required. However, in addition to antipsychotics, antiparkinsonian drugs, antidepressants, or antianxiety drugs may also have to be concomitantly used, therefore these drugs' efficacy and adverse reactions should be considered and adjusted to continue treatment.^6^

Positive symptoms of schizophrenia often respond well to antipsychotics, while negative symptoms do not, and cognitive deficits often remain treatment‐resistant.^7^ In addition, cognitive impairment and negative symptoms are the leading causes of reduced quality of life and impaired daily functioning in patients with schizophrenia. The rate of schizophrenia patients who are refractory to treatment is reported as one‐fifth to one‐half,^8^ and clozapine is reported to be associated with a positive response; however, tolerance to clozapine is one of the clinical issues for treatment resistance in schizophrenia.^9^

In 2005, Iwasaki et al. reported a clinical evaluation of yokukansan for treating cognitive impairment.^10^ The usefulness of yokukansan in the treatment of psychiatric symptoms surrounding dementia drew global attention, and it has been widely used in clinical practice in Japan. Yokukansan is a traditional Japanese herbal medicine product consisting of seven kinds of crude drugs (Atractylodis Lanceae Rhizome, Poria Sclerotium, Cnidium Rhizome, Uncaria Hook, Japanese Angelica Root, Bupleurum Root, and Glycyrrhizae) and is used for the treatment of various symptoms (neurosis, insomnia, night crying in children, and peevishness in children) in patients with a delicate constitution and nervousness. Yokukansan suppresses glutamate release,^11^ corrects glutamate uptake,^12^ downregulates serotonin 2A receptors,^13^ and stimulates serotonin 1A receptors.^14^ Nonclinical studies have shown that yokukansan has anxiolytic‐like effects, inhibits aggression, and improves sleep disorders by modulating the effects of these neurotransmitters.

We previously reported the effects of yokukansan on schizophrenia in open‐label or double‐blind studies as an add‐on to existing treatments or as monotherapy.^15^, ^16^, ^17^, ^18^ In all studies, improvement effects were determined, indicating the potential of adjuvant treatment for treatment‐resistant schizophrenia (TRS). We have already conducted a double‐blind, placebo‐controlled study with a 4‐week follow‐up period and reported that yokukansan showed a tendency to be superior to placebo in the reduction of the Positive and Negative Syndrome Scale (PANSS) scores in TRS, but the difference was not statistically significant.^17^

Four weeks of treatment with yokukansan was considered too short to observe an essential effect of yokukansan, which might need longer administration. Therefore, the present study aimed to evaluate the efficacy and safety of yokukansan as an add‐on pharmacotherapy for clinical symptoms in patients with TRS over a 12‐week period. The PANSS was used as the primary endpoint in accordance with previous studies.

## MATERIAL AND METHODS

### Patients

Patients with schizophrenia presenting to 10 psychiatric hospitals in Japan were enrolled based on the following inclusion and exclusion criteria. The inclusion criteria were (1) patients with a Diagnostic and Statistical Manual of Mental Disorders, 4th edition, text revision (DSM‐IV‐TR) diagnosis of schizophrenia who showed no improvement in their condition for 3 months or longer despite treatment with antipsychotics; (2) inpatients; (3) patients of any sex; (4) patients who were between 18 and 70 years of age at the time of obtaining their consent; (5) patients for whom written consent was obtained before the study examination and observation; (6) patients who had 60 points or more of a total PANSS score at the start of Stage 1 (4 weeks before enrollment/allocation); and (7) patients whom the research physician judged to be able to comply with the criteria. The exclusion criteria were (1) at the end of Stage 1 (enrollment/allocation), the total PANSS score had decreased by at least five points from the start of Stage 1 (4 weeks before enrollment/allocation); (2) patients who were comatose; (3) patients who were under the strong influence of a central nervous system depressant, such as a barbiturate derivative or an anesthetic; (4) patients who were on adrenaline treatment; (5) patients who had a history of hypersensitivity to the study drug (yokukansan); (6) patients who had a history of hypokalemia; (7) patients who had any other Axis I psychiatric disorder besides schizophrenia, and the main diagnosis was not schizophrenia; (8) patients who had borderline personality disorder; (9) mental retardation or brain organic disorder; (10) patients who currently had substance abuse or substance dependence according to DSM‐IV‐TR diagnostic criteria; (11) patients who were complicated by a malignant tumor; (12) patients complicated by severe heart disease, liver disease, kidney disease, blood disease, lung disease, and other diseases judged to affect their lives (assessed according to the degree to which patients had a significant difficulty in performing daily activities and required treatment); (13) patients in whom herbal medicines were taken within 4 weeks prior to enrollment/allocation; (14) pregnant women, women who may be pregnant, women who wish to become pregnant, or women who are breastfeeding; (15) patients in whom oral administration was not possible; (16) patients who participated in other clinical trials or clinical studies within 3 months prior to enrollment/allocation; and (17) other patients judged inappropriate by the research physician.

### Study design

The study was conducted between January 2014 and September 2016 as an investigator‐initiated study. This multicenter, double‐blind, randomized, placebo‐controlled, centrally randomized study was approved by the Medical Research Ethics Committee of the Shimane University School of Medicine and the ethics committees of the participating institutions.

**Figure 1:**
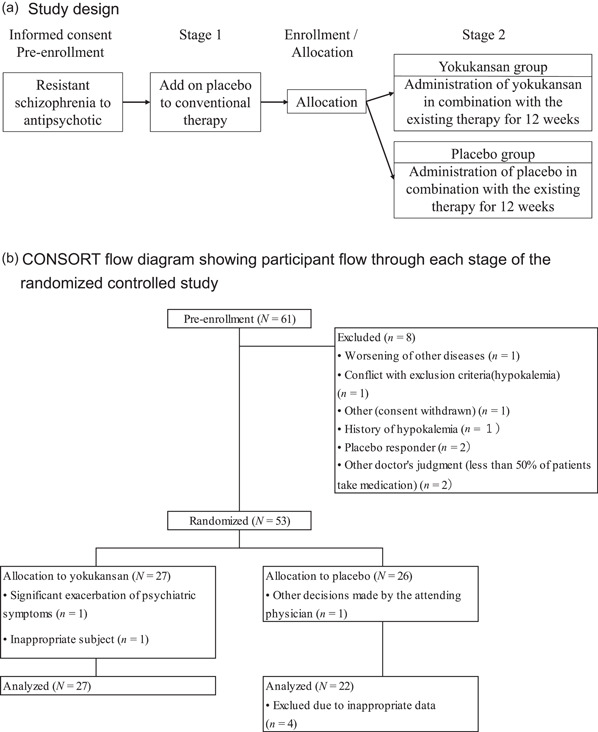
(a) Study design and (b) CONSORT flow diagram showing participant flow through each stage of the randomized controlled study.

The study procedure is illustrated in Figure 1a. Provisional enrollment was initiated on obtaining consent from the patient via the web system, and the evaluation of PANSS was initiated with the administration of the placebo in combination with the existing therapy. To ensure that there was not a decrease of more than five points, the PANSS was again evaluated 4 weeks after placebo administration. The study director registered patient information via the internet. If the information did not meet the eligibility criteria, the patient was judged to be ineligible and not registered. The allocation information was immediately returned to the study investigator if it was accurate. The study investigator followed the allocation instructions (patient enrollment number) and started administering the study drug with the relevant patient number. The allocation table was prepared in advance by the investigator for drug allocation in a 1:1 ratio of the study drug and placebo using the block randomization method, considering the institute.

### Study drug

Tsumura Yokukansan Extract Granules for Ethical Use (Tsumura & Co.) and a placebo were used. Yokukansan contains 3.25 g of dried aqueous extracts from seven kinds of Chinese herbal medicine out of 7.5 g of the product. In this study, 7.5 g was divided into two to three doses per day and orally administered before or between meals. The concurrent use of all herbal medicines was prohibited during the study period (from the time of provisional enrollment to the end of the study). Antipsychotics, antiparkinsonians, anxiolytics, sleep‐inducing drugs, antidepressants, mood stabilizers, and psychostimulants may have been used concomitantly during the treatment period, but no changes in dosage or administration were made, and no additive drugs were used. If an adverse event or other unavoidable event occurred, we allowed changes in the treatment at the discretion of the study investigator. No restrictions were placed on the drugs used to treat the complications, except for herbal medicines.

### Outcome measures

The efficacy observation items were the PANSS, Clinical Global Impressions‐Severity Scale (CGI‐S), Global Assessment of Functioning Scale (GAF), and Drug Induced Extra‐Pyramidal Symptoms Scale (DIEPSS), which were assessed 4 weeks before enrollment/allocation, at enrollment/allocation (before administration), and at 4, 8, and 12 weeks after administration. The primary endpoint was the change in PANSS score from preadministration to 12 weeks after administration, and other items were the secondary endpoints. The types and daily doses of antipsychotics, antiparkinsonian drugs, anxiolytics, sleep inducers, antidepressants, mood stabilizers, and psychostimulants were also recorded.

Safety investigations included aspartate aminotransferase (AST), alanine aminotransferase (ALT), alkaline phosphatase (ALP), γ‐glutamyl transpeptidase (γ‐GTP), and total bilirubin and electrolytes (Na, K, and Cl) as biochemical blood tests, and blood pressure, heart rate, and the presence or absence of peripheral edema as physical examinations 4 weeks before enrollment/allocation (before administration) and at 4, 8, and 12 weeks after administration. Adverse events were investigated throughout the study for the presence or absence of adverse drug reactions, symptoms, and severity.

### Statistical analysis

Because this was a small exploratory study using a placebo for 12 weeks as a preliminary step in determining the long‐term efficacy and safety of yokukansan for schizophrenia, we did not perform an exact sample size estimation, and a feasible number of patients was set.

We determined the amount of change in PANSS scores before and after 4, 8, and 12 weeks of treatment for each participant and performed a comparison between groups using Bonferroni multiple comparisons. We also calculated the difference between the groups in terms of the mean change in PANSS score and its 95% confidence interval. Analyses for secondary endpoints were performed similarly to those for primary endpoints. The differences at each visit between the groups were analyzed using Bonferroni correction, considering multiplicity. All statistical tests were two‐sided, and *P* < 0.05 was considered statistically significant. Analyses were performed using SAS version 9.4 (SAS Institute).

## RESULTS

### Patient disposition and characteristics

PANSS was evaluated in 61 consenting patients during provisional enrollment (4 weeks before enrollment/allocation). During the period before enrollment/allocation, eight patients were excluded and 53 patients were allocated to the yokukansan group (27 patients) and the placebo group (26 patients). After enrollment, two patients in the yokukansan group and one patient in the placebo group discontinued the study but were considered eligible for analysis. However, four patients in the placebo group were considered unsuitable for analysis due to inappropriate data, therefore efficacy was evaluated in 27 patients in the yokukansan group and 22 in the placebo group. Safety was evaluated in 27 patients in the yokukansan group and 26 in the placebo group. The CONSORT flow diagram showing participant flow through each stage of the randomized controlled study is presented in Figure 1b.^19^ Patient characteristics at enrollment in the efficacy evaluation population are shown in Table 1. Concurrent antipsychotic drugs were evaluated, as shown in Table 2. Overall, typical antipsychotic drugs were used by 65.3% of patients, atypical antipsychotic drugs by 81.6%, and antianxiety/hypnotic drugs by 10.2%. There were no changes in dosage or the administration of additional drugs during the study.

**Table 1:** Patient characteristics (efficacy analysis set).

Characteristic		Yokukansan group (*N* = 27)		Placebo group (*N* = 22)		*P*‐value
Mean age, years		59.6 ± 7.5		56.7 ± 8.8		0.223
Sex, *n* (%)						
Male		7 (25.9)		17 (77.3)		<0.001
Female		20 (74.1)		5 (22.7)		
Mean height, cm		158.6 ± 7.9		164.2 ± 8.5		0.022
Mean weight, kg		53.2 ± 11.7		57.9 ± 10.7		0.155
Mean BMI, kg/m^2^		21.04 ± 3.68		21.49 ± 3.94		0.679
Mean length of stay, months		102.7 ± 84.2		189.6 ± 177.8		0.044
Mean schizophrenia duration, months		369.7 ± 148.7		392.0 ± 153.3		0.609
Mean treatment duration, months		331.8 ± 167.2		372.5 ± 163.8		0.396
Complications, *n* (%)						
None		3 (11.1)		0 (0.0)		0.242
Yes		24 (88.9)		22 (100.0)		
Constipation		20 (74.1)		17 (77.3)		
Parkinsonism		11 (40.7)		10 (45.5)		
Hypertension		5 (18.5)		3 (13.6)		
Insomnia		3 (11.1)		5 (22.7)		
Chronic gastritis		4 (14.8)		3 (13.6)		
Diabetes mellitus		4 (14.8)		1 (4.5)		
Past medical history, *n* (%)						
None		25 (92.6)		22 (100.0)		0.495
Yes		Yes		2 (7.4)		
Brain tumor operation		1 (3.7)				
Epilepsy		1 (3.7)				
Cerebral hemorrhage		1 (3.7)				
Intestinal obstruction		1 (3.7)				

*Note*: The *P*‐values of *t*‐test or Fisher's test are shown. Complications in more than five patients in both groups are listed.

Abbreviation: BMI, body mass index.

**Table 2:** Existing treatments (efficacy analysis set).

Treatment	Yokukansan group (*N* = 27)		Placebo group (*N* = 22)	
	*n*	%	*n*	%
Serotonin and dopamine antagonists	16	59.3	14	63.6
Butyrophenone antipsychotics	10	37.0	10	45.5
Phenothiazine antipsychotics	8	29.6	9	40.9
Multiacting receptor targeted antipsychotics	10	37.0	8	36.4
Phenothiazine antipsychotics/barbiturates/antihistamines	9	33.3	6	27.3
Benzodiazepines	3	11.1	2	9.1
Dopamine receptor partial agonists	4	14.8	1	4.5
Benzamide antipsychotics	1	3.7	1	4.5
Nonbenzodiazepine hypnotics	1	3.7	0	0.0

### Primary endpoint: change in PANSS scores

**Figure 2:**
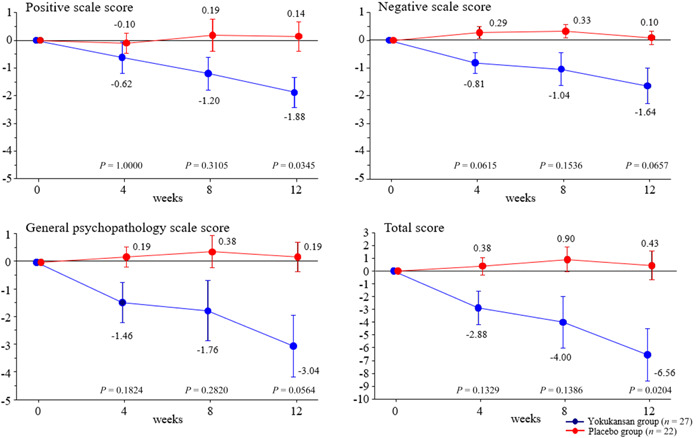
Change in each Positive and Negative Symptom Scale score from pretreatment. Upper left, positive scale score; upper right, negative scale score; lower left, general psychopathology scale score; lower right, total score. Red line, placebo group; blue line, yokukansan group. The figures represent the mean scores at 4, 8, and 12 weeks. In the yokukansan group, the number of patients was 26, 25, and 25 at 4, 8, and 12 weeks, respectively, while the placebo group consisted of 21 patients. Circles with numbers indicate mean changes from allocation and standard error, respectively. *P*‐values between groups were calculated by Bonferroni correction.

The changes with time in the PANSS scores from pretreatment to week 12 are shown in Table 3 and Figure 2. The positive, negative, and general psychopathology PANSS scores decreased in the yokukansan group but not in the placebo group. Positive PANSS scores decreased from pretreatment to week 12 by 1.88 ± 0.54 (mean ± standard error) in the yokukansan group and increased by 0.14 ± 0.54 in the placebo group, with a significant difference in the change at 12 weeks between groups (*P* = 0.0345; Bonferroni multiple comparison). Negative PANSS scores decreased by 1.64 ± 0.64 in the yokukansan group and increased by 0.10 ± 0.24 in the placebo group, with no significant difference between groups (*P* = 0.0657). The general psychopathology PANSS scores decreased by 3.04 ± 1.12 in the yokukansan group and increased by 0.19 ± 0.55 in the placebo group, with no significant difference between groups (*P* = 0.0564). The total PANSS decreased by 6.56 ± 2.05 in the yokukansan group and increased by 0.43 ± 1.13 in the placebo group, with a significant difference between groups (*P* = 0.0204).

**Table 3:** Results of efficacy endpoints (efficacy analysis set).

Evaluation parameter		Yokukansan group (*N* = 27)								Placebo group (*N* = 22)							
		At allocation		After 4 weeks		After 8 weeks		After 12 weeks		At allocation		After 4 weeks		After 8 weeks		After 12 weeks	
PANSS																	
Number of patients		27		26		25		25		22		21		21		21	
Positive scale		25.1 ± 1.3		24.7 ± 1.3		23.9 ± 1.4		23.2 ± 1.4		27.2 ± 1.8		27.3 ± 1.7		27.6 ± 1.9		27.5 ± 1.9	
Negative scale		28.8 ± 1.5		28.0 ± 1.6		27.3 ± 1.7		26.7 ± 1.6		27.6 ± 1.6		27.8 ± 1.7		27.9 ± 1.7		27.6 ± 1.7	
General psychopathology scale		52.0 ± 2.5		50.3 ± 2.7		49.4 ± 2.9		48.1 ± 2.8		53.6 ± 3.3		54.2 ± 3.4		54.4 ± 3.4		54.2 ± 3.4	
Total scale		105.9 ± 4.8		103.0 ± 5.4		100.6 ± 5.7		98.0 ± 5.5		108.4 ± 6.2		109.3 ± 6.3		109.8 ± 6.5		109.3 ± 6.5	
CGI‐S		5.11 ± 0.19		5.04 ± 0.20		4.96 ± 0.22		4.88 ± 0.22		5.32 ± 0.23		5.43 ± 0.24		5.48 ± 0.24		5.48 ± 0.24	
GAF		29.1 ± 1.61		30.5 ± 1.94		31.6 ± 2.20		33.3 ± 2.28		27.0 ± 1.94		26.5 ± 2.06		25.9 ± 1.94		25.9 ± 2.10	
DIEPSS																	
Gait		1.11 ± 0.19		1.15 ± 0.21		1.16 ± 0.22		1.16 ± 0.21		1.14 ± 0.23		1.20 ± 0.24		1.20 ± 0.24		1.20 ± 0.24	
Bradykinesia		1.41 ± 0.25		1.35 ± 0.25		1.24 ± 0.24		1.28 ± 0.23		1.45 ± 0.23		1.52 ± 0.22		1.52 ± 0.22		1.52 ± 0.22	
Sialorrhea		0.30 ± 0.12		0.31 ± 0.12		0.28 ± 0.15		0.28 ± 0.14		0.73 ± 0.23		0.76 ± 0.24		0.76 ± 0.24		0.76 ± 0.24	
Muscle rigidity		0.26 ± 0.14		0.27 ± 0.14		0.24 ± 0.14		0.24 ± 0.13		0.36 ± 0.17		0.38 ± 0.18		0.38 ± 0.18		0.38 ± 0.18	
Tremor		0.78 ± 0.19		0.73 ± 0.18		0.76 ± 0.18		0.72 ± 0.17		0.86 ± 0.21		0.90 ± 0.22		0.95 ± 0.21		0.90 ± 0.22	
Akathisia		0.19 ± 0.12		0.15 ± 0.09		0.16 ± 0.12		0.16 ± 0.12		0.23 ± 0.15		0.24 ± 0.15		0.24 ± 0.15		0.24 ± 0.15	
Dystonia		0.11 ± 0.06		0.12 ± 0.06		0.08 ± 0.06		0.08 ± 0.06		0.09 ± 0.09		0.10 ± 0.10		0.10 ± 0.10		0.10 ± 0.10	
Dyskinesia		0.37 ± 0.19		0.35 ± 0.17		0.36 ± 0.18		0.36 ± 0.18		0.18 ± 0.11		0.19 ± 0.11		0.24 ± 0.14		0.19 ± 0.11	
Overall severity		1.44 ± 0.22		1.46 ± 0.23		1.32 ± 0.21		1.28 ± 0.20		1.59 ± 0.21		1.67 ± 0.21		1.67 ± 0.21		1.67 ± 0.21	

*Note*: Data are expressed as mean ± standard error.

Abbreviations: CGI‐S, Clinical Global Impression‐Severity Scale; DIEPSS, Drug‐Induced Extrapyramidal Symptom Scale; GAF, Global Assessment Scale of Function; PANSS, Positive and Negative Symptom Scale.

### Secondary endpoints

The changes in the CGI‐S, GAF, and DIEPSS scores from pretreatment to 12 weeks after administration in each group are shown in Table 3. CGF‐S tended to decrease in the yokukansan group and increase in the placebo group, but the changes did not differ significantly. GAF also showed similar changes; however, the changes after 8 and 12 weeks of treatment were significantly higher in the yokukansan group (*P* < 0.06, *P* < 0.02). There were no significant changes in DIEPSS from pretreatment to 12 weeks of treatment in either group for any of the nine components.

### Safety

The adverse events following the administration of yokukansan or placebo are shown in Table 4. All adverse events observed in this study were adverse drug reactions that could not be ruled out as causally related to the drug. Adverse drug reactions were reported in six of 27 patients (22.2%) in the yokukansan group and five of 26 patients (19.2%) in the placebo group, all of which were nonserious. Adverse drug reactions were reported as follows: in the yokukansan group, hepatic dysfunction (*N* = 2), hypokalemia (*N* = 1), depressive state (*N* = 1), insomnia, anxiety, and bad mood (*N* = 1), and osteoporosis (*N* = 1); and in the placebo group, hepatic dysfunction (*N* = 1), hypokalemia (*N* = 1), AST elevation (*N* = 1), low morale and moodiness (*N* = 1), and left shoulder periarthritis (*N* = 1). Two patients in the yokukansan group discontinued the study due to adverse drug reactions (hypokalemia and depression), but none did in the placebo group.

**Table 4:** List of adverse events of study intervention by 12 weeks (safety analysis set).

Group	Event name	Cases
Yokukansan group	Hepatic dysfunction	2
(*N* = 27)	Hypokalemia	1
	Depressive state	1
	Insomnia, anxiety, and bad mood	1
	Osteoporosis	1
Placebo group	AST elevation	1
(*N* = 26)	Hepatic dysfunction	1
	Hypokalemia	1
	Low morale and bad mood	1
	Left shoulder periarthritis	1

Abbreviation: AST, aspartate aminotransferase.

## DISCUSSION

From the results of this study, in patients with TRS treated with multiple antipsychotics, there were no changes in psychiatric symptoms after placebo treatment; however, those treated with yokukansan after 12 weeks showed significant improvement, mainly in the positive PANSS scores of psychiatric symptoms. The adverse reaction rate was not significantly different between groups: two patients in the yokukansan group discontinued the study due to adverse reactions, but none did in the placebo group.

The etiology of schizophrenia remains unknown, but multipronged investigations including genetics, neurophysiology, and neurobiology suggest that many factors related to various clinical symptoms are involved. It has been proposed that positive symptoms of schizophrenia involve altered dopamine neurotransmission in the mesolimbic system, and negative symptoms of schizophrenia involve the dopaminergic and glutamatergic hypotheses.^20^ Increased presynaptic dopamine synthesis has been linked to the pathogenesis of schizophrenia.^21^ The involvement of other neurotransmitters in the brain has also been investigated, leading to the development and clinical application of drugs with diverse mechanisms of action, such as antipsychotics.

In our previous study about the efficacy of yokukansan in patients with TRS, the positive and negative PANSS scores decreased significantly after 4 weeks of treatment with yokukansan added to existing therapies in an open‐label study.^15^ Another open‐label study evaluated the effect of yokukansan monotherapy for very late‐onset schizophrenia‐like psychosis for 4 weeks, significantly reducing total, positive, negative, and general psychopathology PANSS scores.^16^ These results indicate that yokukansan is effective for positive, negative, and general psychopathology symptoms evaluated by the PANSS, which can be understood as the benefits of the multiple pharmacological effects of yokukansan.

In contrast, in our double‐blind placebo‐controlled study of TRS, the total PANSS score, and PANSS subscale scores for positive, negative, and general psychopathology symptoms in patients treated with yokukansan tended to be better than those with placebo over 4 weeks, and significant improvement was observed in the yokukansan group compared to the placebo group for lack of spontaneity and flow of conversation, tension, and poor impulse control.^17^ In another prior double‐blind study conducted by our team on TRS using PANSS five‐factor analysis, the administration of yokukansan for 4 weeks resulted in significant improvements in excitement/hostility factor PANSS scores compared to placebo. However, there were no significant differences in PANSS total, depression/anxiety, cognition, or positive or negative factors.^18^

The results of our previous 4‐week short‐term administration studies of yokukansan support the clinical utility of yokukansan in schizophrenia, but one factor to be considered was the evaluation period because there were endpoints that did not show a clear difference from placebo. We therefore decided to evaluate the effects of yokukansan in a 12‐week, double‐blind, placebo‐controlled study focused on TRS because, in the clinical setting, we often observed the therapeutic effect of yokukansan for over 4 weeks of administration. In the current study, the reductions in the total and positive PANSS scales with yokukansan were significantly superior to those with placebo. Yokukansan tended to decrease scores over time until at least 12 weeks later. This indicates that the effects of yokukansan can be detected even with a short administration of 4 weeks, but a longer observation would be suitable to detect its effect in TRS. Indeed, the results of this study reflected opinions on the benefits of yokukansan experienced in actual clinical practice.

In the present study, there was no change in the dosage or administration of the antipsychotics used before the study, therefore there was no change in the dosage of the antipsychotics used concurrently. Generally, schizophrenia is thought to develop with dysfunction of multiple neural functions, and atypical antipsychotics acting on dopamine, serotonin, or cholinergic receptors are used along with typical antipsychotics, whose main action is dopamine receptor inhibition. Drugs in both classes were selected according to symptoms in the current study. Small but robust differences were reported in the efficacy of individual drugs, and antipsychotics differed substantially in adverse reactions among patients.^8^ They are not always effectively controlled by either drug, and avoiding drug‐related problems is also important, necessitating more effective and safer treatments.^22^ Yokukansan appears to be a treatment option for refractory schizophrenia.

### Limitation

The changes in PANSS scores in this study were not as great as those in the open‐label study, and differences in patient populations are a factor that must be considered. In addition, the dosage and administration of yokukansan also need to be systematically investigated to determine the appropriate dosage and administration for the approved indications.

## CONCLUSIONS

While the treatment of schizophrenia traditionally relies on the dopaminergic hypothesis of the disease, innovative approaches have surfaced, exploring novel signaling mechanisms. Schizophrenia is a complex, multifactorial disease, and based on current knowledge, not all symptoms of the disease can be treated with a single targeted drug, therefore individualized treatment is necessary for each patient. Since yokukansan is very safe and has diverse pharmacological effects, it is necessary to evaluate its clinical value through further investigation as a treatment that can be used in combination with Western medicine.

## AUTHOR CONTRIBUTIONS

Jun Horiguchi contributed to the manuscript writing. Rei Wake, Haruo Seno, Tsuyoshi Miyaoka, and Ken Inoue contributed to the collection of the data. Kenta Murotani contributed to the data analysis. All the authors have read and approved the final manuscript.

## CONFLICT OF INTEREST STATEMENT

Kenta Murotani received a research grant as the chief of statistical analysis from Tsumura & Co. The other authors declare no conflict of interest.

## ETHICS APPROVAL STATEMENT

This multicenter, double‐blind, randomized, placebo‐controlled, centrally randomized study was approved by the Medical Research Ethics Committee of the Shimane University School of Medicine and the ethics committees of the participating institutions.

## PATIENT CONSENT STATEMENT

Written informed consent was obtained from all participants included in the analyses.

## CLINICAL TRIAL REGISTRATION

The study was registered with the UMIN Clinical Trials Registry before patient enrollment (Study ID: UMIN 000015709).

## Supporting information


**Supporting Information**: CONSORT CHECKLIST.

## Data Availability

The data used to support the findings of this study are available from the corresponding author upon reasonable request.
